# Persistence Infection of TGEV Promotes *Enterococcus faecalis* Infection on IPEC-J2 Cells

**DOI:** 10.3390/ijms24010450

**Published:** 2022-12-27

**Authors:** Zhenzhen Guo, Chenxin Zhang, Jiajun Dong, Yabin Wang, Hui Hu, Liying Chen

**Affiliations:** 1 College of Veterinary Medicine, Henan Agricultural University, Zhengzhou 450002, China; 2 College of Animal Science and Technology, Guangxi University, Nanning 530004, China; 3 Henan Key Laboratory of Animal Food Safety, Zhengzhou 450002, China

**Keywords:** TGEV, EMT, *E. faecalis*, bacterial-viral co-infection

## Abstract

Transmissible gastroenteritis virus (TGEV) is a coronavirus causing diarrhea with high incidence in swine herds. Its persistent infection might lead to epithelial-mesenchymal transition (EMT) of swine intestinal epithelial cells, followed by subsequent infections of other pathogens. *Enterococcus faecalis* (*E. faecalis*) is a member of the enteric microorganisms and an opportunistic pathogen. There is no report of secondary *E. faecalis* infection to TGEV, even though they both target to the intestinal tracts. To investigate the interactions between TGEV and *E. faecalis*, we set up an in vitro infection model by the swine IPEC-J2 cells. Dynamic changes of cell traits, including EMT and cell motility, were evaluated through qPCR, Western blot, electronic microscopy, scratch test, Transwell migration test and invasion test, respectively. The adhesion and invasion tests of *E. faecalis* were taken to verify the impact of the preceding TGEV infection. The cell morphology and molecular marker evaluation results showed that the TGEV persistent infection induced EMT on IPEC-J2 cells; increased cellular motility and invasion potential were also observed. Spontaneously, the expression levels of fibronectin (FN) and the membrane protein integrin-α5, which are dominant bacterial receptors on IPEC-J2 cells, were upgraded. It indicated that the bacteria *E. faecalis* adhered to IPEC-J2 cells through the FN receptor, and then invaded the cells by binding with the integrin-α5, suggesting that both molecules were critical for the adhesion and invasion of *E. faecalis* to IPEC-J2 cells. Additionally, it appeared that *E. faecalis* alone might trigger certain EMT phenomena, implying a vicious circle might occur. Generally, bacterial and viral co-infections are frustrating yet common in both human and veterinary medicines, and our observations on enteric TGEV and *E. faecalis* interactions, especially the diversity of bacterial invasion strategies, might provide new insights into the mechanisms of *E. faecalis* pathogenicity.

## 1. Introduction

Transmissible gastroenteritis virus (TGEV) is a member of the *Coronaviridae Coronavirus* family and can cause an acute and highly contagious viral disease of swine transmissible gastroenteritis. A TGEV infection is often followed by secondary bacterial invasions, such as *Enterotoxigenic Escherichia coli enteritis* [1], *Enterotoxigenic Escherichia coli K88* [2], and porcine epidemic diarrhea virus [3], but there has been no report of *Enterococcus faecalis (E. faecalis)* as the secondary pathogen. *E. faecalis* is a gram-positive, facultatively anaerobic coccus, which usually occurs in pairs or short chains with no spore nor pod, and is negative in catalase or oxidase [4,5]. As an opportunistic pathogen, *E. faecalis* becomes invasive when the host enteritis occurs, aggravating the disease process [6]. The mechanisms of enterococcal infection are adhesion and invasion. Several molecules may assist bacterial adhesion and invasion to host cells through identifying different elements and extracellular matrix components on the surface of the host cells, such as collagen, laminin, elastin acid, hyaluronic acid, and polysaccharide [7,8].

While TGEV and *E. faecalis* both occupy the intestinal tract, they gain the potential to interact, thus promoting disease development. Clinically, TGEV-infected pigs are often secondary to other pathogens, suggesting that TGEV infection might enhance the invasive effect of *E. faecalis* on the intestinal cells. However, because of its commensal trait, *E. faecalis* is always free of charge of any intestinal problems, though it actually can be lethal. In this study, however, *E. faecalis* was found to be pathogenic, incubated with TGEV-infected cells, which is more likely to adhere and invade. Since viral infection leads to activation of intracellular signaling pathways to Epithelial-mesenchymal transition (EMT) [9], we intuitively infer that TGEV can also induce EMT, and interacts with *E. faecalis* through it.

The term EMT originated from Elizabeth Hay’s observations in 1980 describing phenotypic changes from epithelial to mesenchymal cells in primitive stripes of chicken embryos [10]. EMT is natural in organisms, and has been widely studied in the field of human medicine, especially cancer research. Multiple pathological processes, such as renal fibrosis and tumor metastasis, are associated with EMT [11,12]. Typically, EMT results in the disintegration of cell—cell connections, the reconstruction of actin cytoskeletal, and inducing contractile proteins and nonmotile epithelial cells into separate, motile, and aggressive mesenchymal phenotypic cells [13,14,15]. It has been found that bacterial products such as LPS [16], flagellin, and Muramyl dipeptide (MDP) [17] can induce EMT, and some Gram-negative bacteria such as *Escherichia coli* [18] and *Helicobacter pylori* [19] can also induce EMT. Three transcription factor families, i.e., the Snail family, the ZEB family, and the bHLH family, mediate the regulation of EMT transcription, including inhibiting the expression of epithelial marker genes and activating the expression of mesenchymal genes [15]. EMT appears to be the result that growth-factor-induced signaling pathways affect epithelial integrity and target downstream transcriptional regulators to regulate epithelial-to-mesenchymal gene expression, such as the TGF-β [20], Wnt [21], and Notch signaling pathways [22].

Here, we sought to identify the consequence and mechanism of the interactions between TGEV and *E. faecalis*. Moreover, we look forward to providing an idea of the interaction mechanisms of TGEV and *E. faecalis*, which will not only help to explain how TGEV assists other pathogens to invade the cells but draws attention to considering the hazard of *E. faecalis* as well. In this research, the changes of cell characteristics, including EMT and moveability, were evaluated through morphological observation, the scratch test, the Transwell migration and invasion tests. The effects of the TGEV infection on the adhesion and the invasion ratio of *E. faecalis* were determined.

## 2. Results

### 2.1. TGEV Induced EMT in IPEC-J2 Cells

**Figure 1:**
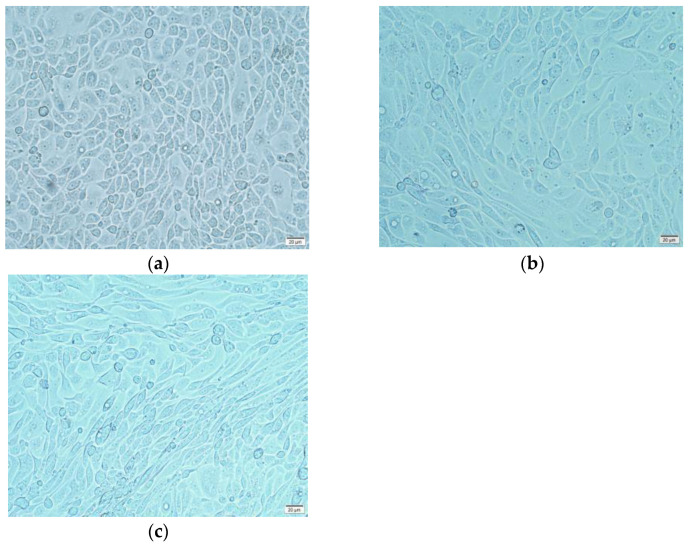
IPEC-J2 cell morphology under 200x inverted microscope. (**a**) Control group: cells were in a typical pebble-stone shape with clear boundary; (**b**) TGEV group: cells were elongated to a spindle shape; (**c**) TGF-β group: cells were elongated to a spindle shape.

EMT is accompanied by unique changes in cell morphology, typically from a cobblestone phenotype to an elongated fibroblast morphology. Besides the negative control group and the TGEV-infected group, the positive control group was set as IPEC-J2 cells incubated with the EMT inducer TGF-β in this and subsequent experiments. The results showed that the cells in the positive group were completely elongated into a spindle shape phenotype similar to fibroblasts (Figure 1b), while normal IPEC-J2 cells presented a typical pebble stone shape with clear boundaries (Figure 1a). In the TGEV-infected group (MOI = 10), only some cells in the 5th generation became fusiform, which was similar to the morphology of the positive control group cells (Figure 1c). This suggested that continuous TGEV infection might induce EMT in IPEC-J2 cells.

**Figure 2:**
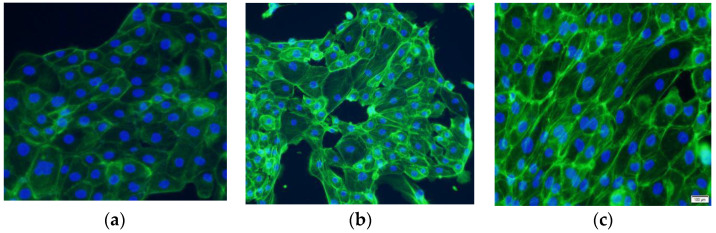
Fluorescence staining of the actin cytoskeleton of IPEC-J2 cells with phalloidine. (**a**) Control group: F-actin was equably distributed; (**b**) TGEV group: F-actin was distributed in bundle; (**c**) TGF-β group: the result was similar to the TGEV group.

The actin cytoskeleton is a highly dynamic structure formed by the aggregation of actin monomers (globular or G-actin) to form F-actin. With EMT developing, cell—cell connections became unstable, and the cytoskeleton started to reconstruct to increase cell motility [23]. As shown in Figure 2, the cytoskeleton was remodeled after EMT had occurred and actin was reprogrammed into the stress fiber F-actin, which may increase cell mobility and aggressiveness. Since phalloidin can specifically bind the dicyclic peptide of F-actin, the actin cytoskeleton was stained by YF-labeled phalloidin to observe the effect of TGEV on the production and distribution of F-actin. The results revealed that F-actin was evenly distributed in the cytoplasm of cells of the negative control group, while in both the positive control and the TGEV-infected group, F-actin notably increased in number and was distributed in bundles.

**Figure 3:**
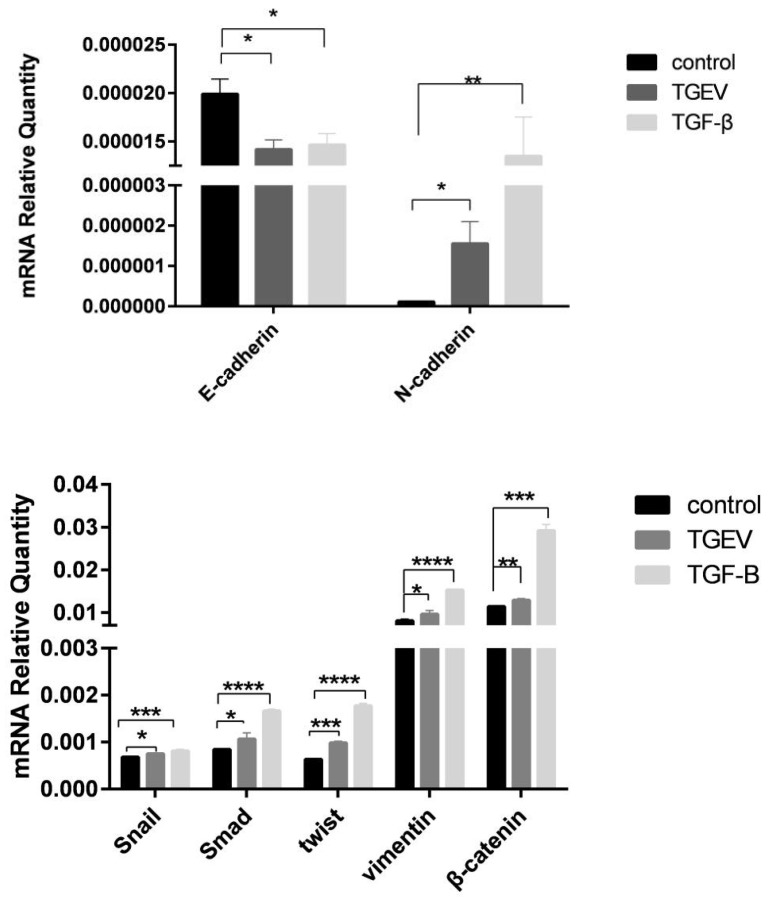
qPCR was used to assess the mRNA expression levels of interstitial markers, including N-cadherin, β-catenin, Smad, Twist, snail and vimentin, and epithelial markers E-cadherin. * *p* < 0.05, ** *p* < 0.01, *** *p* < 0.001, **** *p* < 0.0001.

**Figure 4:**
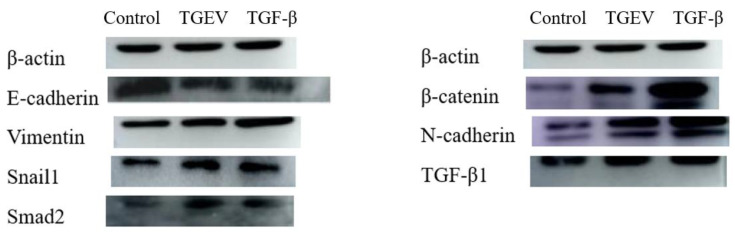
Western blot analysis of EMT markers.

In addition to the phenotypic tests, fluorescence qPCR and Western blot methods were applied to detect the occurrence of EMT and the extent quantification of EMT, further confirmed by transcriptional and protein levels of EMT markers, including E-cadherin, N-cadherin, Vimentin, Twist, snail, β-catenin, and Smad. In the TGF-β group, the mesenchymal cell markers N-cadherin, β-catenin, Smad, Twist, snail, and vimentin presented significantly increased expression levels, whereas the expression of epithelial marker E-cadherin were significantly down-regulated (Figure 3). The TGEV-infected cells showed the same trend as the positive control, indicating that TGEV up-regulated the transcription level of EMT markers at the transcriptional level. Western blot was used to analyze the expression level of EMT marker protein. Using β-actin as an internal reference, the expression levels of E-cadherin in TGEV and TGF-β groups were found to be significantly lower than that in the normal cells. The expression levels of N-cadherin, β-catenin, Smad, Twist, snail, and vimentin were significantly increased, which is consistent with qPCR results, as shown in Figure 4 and Figure A4.

**Figure 5:**
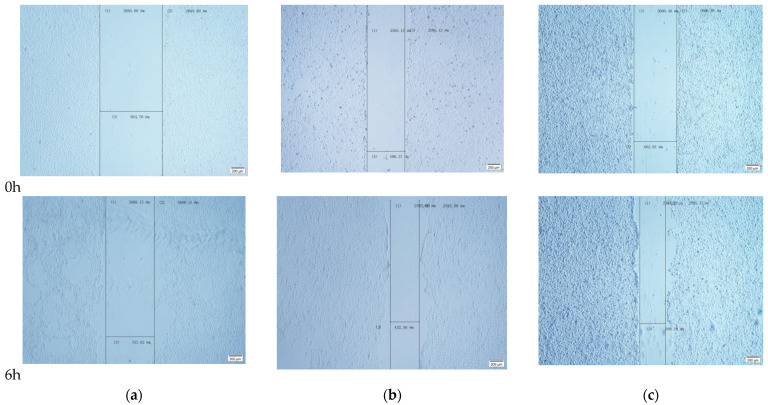
Effects of TGEV infection on cell width. (**a**) Control group; (**b**) TGEV group; (**c**) TGF-β group.

**Figure 6:**
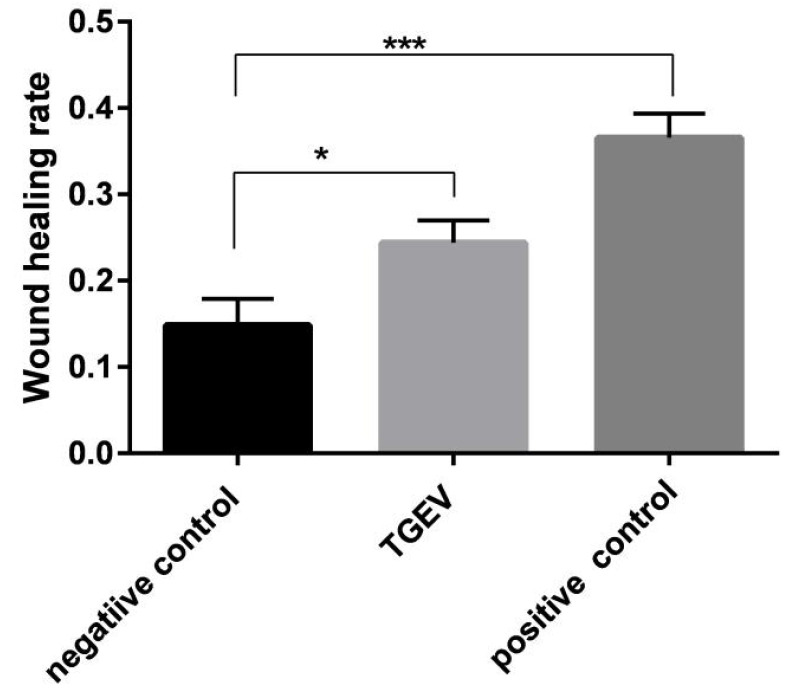
The change of cell scratch healing rate by TGEV infection. * *p* < 0.05, *** *p* < 0.001.

**Figure 7:**
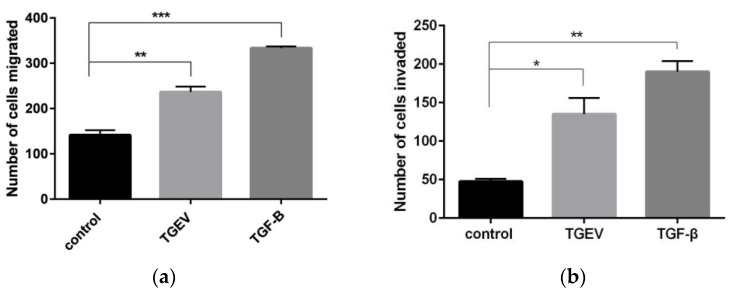
Migratory and invasiveness capacity of IPEC-J2 cells in response to TGEV infection. (**a**) Transwell migration test; (**b**) Transwell invasion test. * *p* < 0.05, ** *p* < 0.01, *** *p* < 0.001.

Aggressiveness of cell movement was assessed through scratch test, Transwell migration and invasion test. A scratch test is to measure the cell plane migration ability. The results show that the cell width of the TGF-β group decreased significantly at 6 h (Figure 5), indicating that TGF-β could improve the rate of intercellular healing; that is, effectively induced cell migration. Compared with the TGF-β group, the cell width of the TGEV-infected cells decreased slightly, but significantly referred to that of the normal cells, and the gap healing rate was significantly increased (Figure 6). Similarly, in Transwell migration and Transwell invasion tests, migratory and invasiveness capability of the IPEC-J2 cells in TGEV and the TGF-β group also increased (Figure 7). These results indicated that TGEV could also induce cell migration, resulting in enhanced cell motility.

### 2.2. TGEV Infection Enhanced the Adhesion of E. faecalis to IPEC-J2 Cells

**Figure 8:**
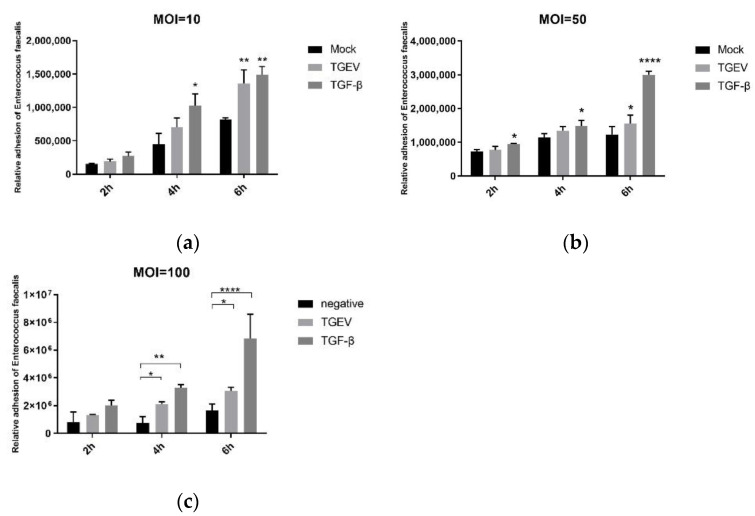
Adhesion of *E. faecalis* to IPEC-J2 cells at different infection rates and time points. (**a**) MOI = 10; (**b**) MOI = 50; (**c**) MOI = 100. * *p* < 0.05, ** *p* < 0.01, **** *p* < 0.0001.

Firstly, TGEV-infected IPEC-J2 cells were continuously cultured to 5th generation, and the pretest and the cell activity test were carried out, which proved that cell activity was above 90% (results not shown). The cells were then incubated with different quantities of *E. faecalis* (MOI = 10, 50, and 100) for different time lengths (2 h, 4 h, and 6 h). After culturing, the adhesion of *E. faecalis* on the IPEC-J2 cells were detected. The results show that both TGEV and TGF-β could promote the adhesion of *E. faecalis*, and bacterial adhesion reached the highest level when the MOI = 100 group was incubated for 6 h (Figure 8).

**Figure 9:**
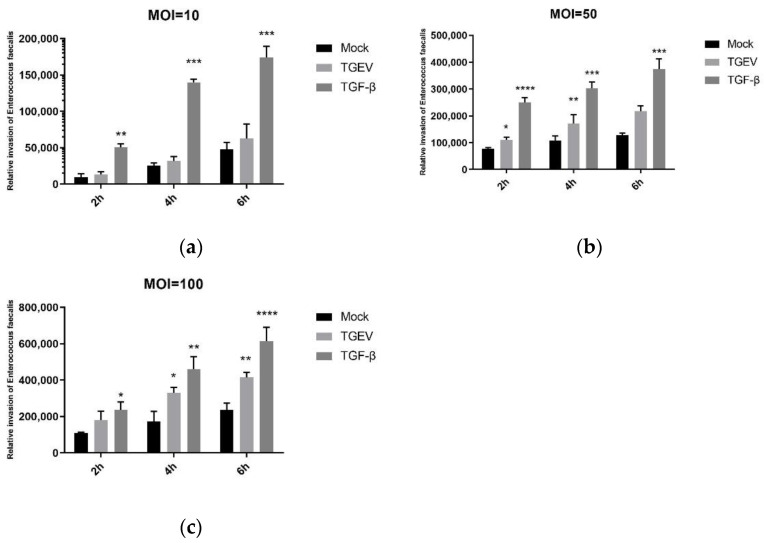
Invasion of IPEC-J2 cells by *E. faecalis* at different infection rates and time points. (**a**) MOI = 10; (**b**) MOI = 50; (**c**) MOI = 100. * *p* < 0.05, ** *p* < 0.01, *** *p* < 0.001, **** *p* < 0.0001.

In addition, the *E. faecalis* invasion was enumerated as well. It showed that the numbers of invaded bacteria were far less than the corresponding number of adhered ones. The *E. faecalis* invading into the IPEC-J2 cells in the TGF-β group were more than those in the control group, but this invasion of *E. faecalis* was significantly promoted with TGEV’s presence (Figure 9). Based on the adhesion and invasion of the bacteria, the optimal concentration and time duration for *E. faecalis* to adhere to IPEC-J2 cells was MOI = 100 and incubated for 6 h, and such conditions were used for subsequent experiments.

**Figure 10:**
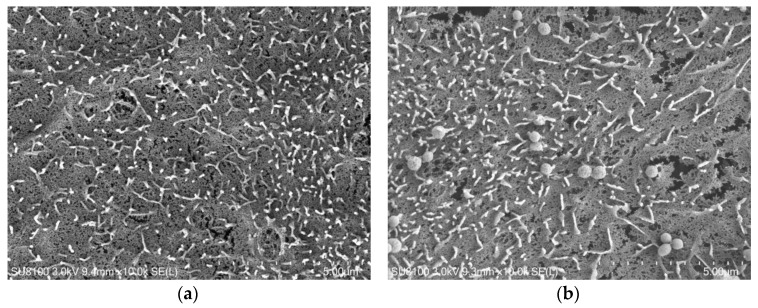

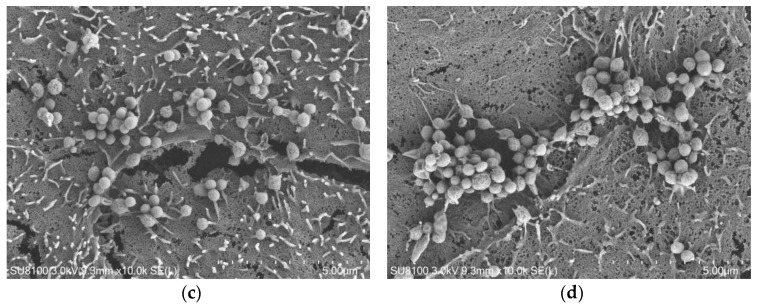
Scanning electron microscope observation on adhesion of *E. faecalis* to IPEC-J2 cells. (**a**) Blank control group; (**b**) Negative control group; (**c**) TGEV group; (**d**) TGF-β group.

To further observe the adhesion of *E. faecalis* to IPEC-J2 cells and its state on the cell surface, field emission scanning electron microscopy was used. The IPEC-J2 cell line as an in vitro model was highly similar to the morphology of intestinal epithelial cells derived from the IPEC-J2 cell line in vivo, with microvilli on its tip. Figure 10 shows that the adhesion trend of *E. faecalis* was the TGF-β group > the TGEV group > the negative control group. In addition, *E. faecalis* also had adhesion ability to normal IPEC-J2 cells. Most of the *E. faecalis* that adhered to the IPEC-J2 cells were in a state of aggregation, and *E. faecalis* could bind to the microvilli on the top of IPEC-J2 cells.

### 2.3. EMT Promoted the Expression of the E. faecalis Receptor and the Migration of E. faecalis

**Figure 11:**
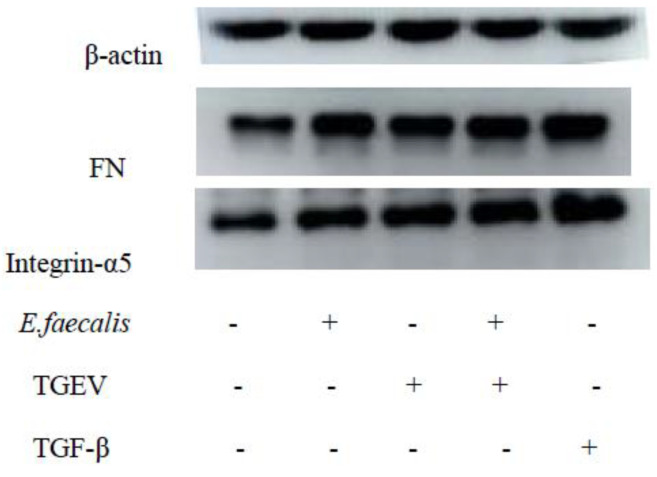
Western blot of *E. faecalis* receptors under different conditions. The first and the fifth groups are the negative and positive control groups.

Bacterial pathogens recognize fibronectin (FN) and then bind to the transmembrane receptor integrin, mediating bacterial adhesion and invasion [8]. The FN-binding protein EfbA was first demonstrated to contribute to the pathogenesis of *E. faecalis* infective endocarditis [24], indicating that FN is also one of the receptors of *E. faecalis*. The IPEC-J2 cells were incubated with *E. faecalis* for 6 h, and the expression changes of FN and integrin-α5 after EMT were analyzed by Western blot. The results showed that both FN and integrin-α5 expressions were up-regulated after EMT, which was even more significant in the co-infection group (Figure 11 and Figure A5).

**Figure 12:**
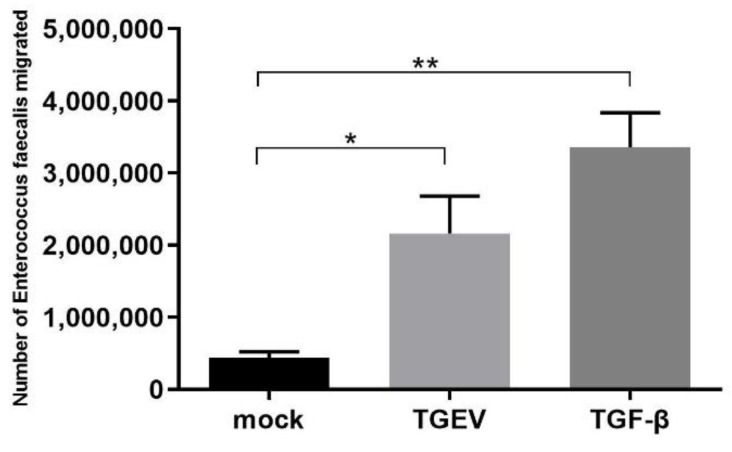
Migration number of *E. faecalis* after infection. * *p* < 0.05, ** *p* < 0.01.

The enhancing effect of EMT on the adhesion of *E. faecalis* was also reflected in the improvement of bacterial mobility. The result of the Transwell bacterial migration experiment showed that the counts of bacterial migration were the highest in the positive control group, followed by that of the TGEV group, and both significantly eclipsed the normal IPEC-J2 cell group (Figure 12). The above results suggested that TGF-β induces EMT in all IPEC-J2 cells, thereby enlarging the intercellular space, while TGEV just induces EMT in some of cells. It also suggests that *E. faecalis* can enter tissues through the enlarged intercellular space, which is a strategy that bacteria use to cross the host barrier.

**Figure A1:**
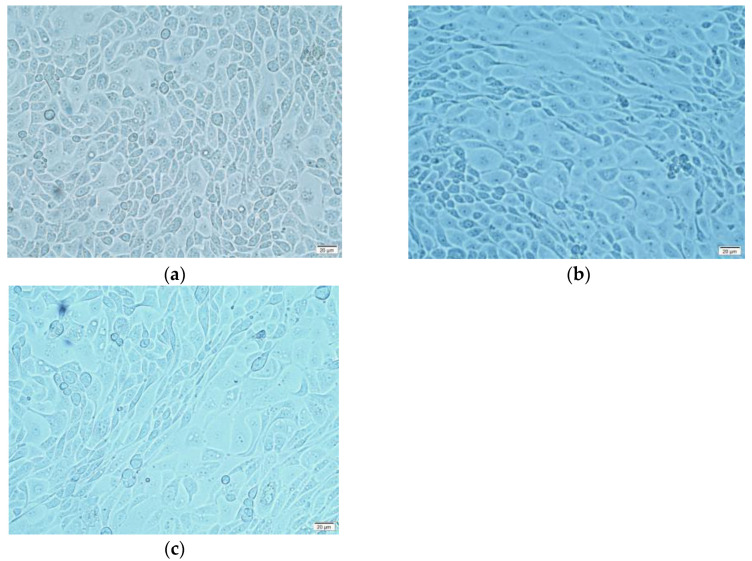
Effect of *Enterococcus faecalis* infection on IPEC-J2 cell morphology. (**a**) Control group; (**b**) *Enterococcus faecalis* group, cells were elongated to a spindle shape; (**c**) TGF-β group.

**Figure A2:**
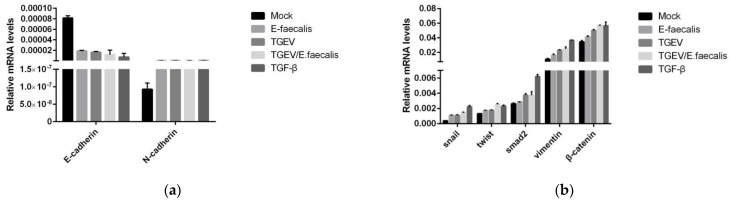
Evaluation of the effect of *Enterococcus faecalis* on the transcription level of EMT markers by RT−PCR. (**a**) Expression levels of E-cadherin and N-cadherin; (**b**) Expression levels of snail, twist, smad2, vimentin and β-catenin.

Additionally, while the expression of FN and integrin-α5 in the TGEV-*E. faecalis* group is higher than that of the TGEV group, the possibility of *E. faecalis* alone triggering EMT was taken into consideration. Further experiments showed that the IPEC-J2 cells incubated with *E. faecalis* (MOI = 100) had typical characteristics of EMT cell morphology (Figure A1) and transcription levels of EMT markers (Figure A2). As for the TGEV-infected cells, the EMT marker levels increased with *E. faecalis* co-existing, which indicated that *E. faecalis* itself could induce EMT, and this might get enhanced when EMT had already occurred.

## 3. Discussion

### 3.1. EMT Occurred after IPEC-J2 Cells Were Infected with TGEV

The epithelial cells form the physical barriers between the organism body and the outside. Under healthy conditions, the epithelial cells are tightly bound to adjacent cells and the underlying basement membrane through various structures, such as adhesion junctions, tight junctions, desmosomes, and hemidesmosomes [25]. However, epithelial cells could be endowed with a high degree of plasticity. During development, fibrogenesis, or tumor progression, they may lose their static phenotypes and acquire migration and invasion behaviors [26]. Growth factors bind to their respective receptors, triggering the activation of multiple signaling pathways that ultimately stimulate core EMT regulators [20]. In cancer, many stem cell pathways, such as Wnt, RAS, Sonic Hedgehog (SHH), and Notch, are overactivated, enabling tumor cells to possess EMT properties. EMT is associated with phenotypic and genotypic changes. Typically, epithelial cells undergoing EMT lose their cobblestone phenotype to obtain an elongated fibroblast morphology [27]. Genetically, the down-regulation of E-cadherin, the down- regulation and translocation of β-catenin from cell membrane to nucleus, and the up-regulation of interstitial markers such as vimentin, fibronectin, and N-cadherin, are frequently detected with EMT occurrence. These changes induce the disconnection of cells and the reconstruction of actin cytoskeleton, and finally drive the non-motile epithelial cells transferring into separate, motile, and aggressive mesenchymal phenotypic cells.

Our study reveals that both the cells of the TGF-β group and the TGEV group shared similar changes in the transcription and expression levels of EMT markers (E-cadherin, N-Cadherin, vimentin, Twist, snail, β-catenin, and Smad). The TGF-β group and the TGEV group cells were also observed to have the phenotypic EMT characteristics under the microscope. Moreover, the scratch test, the Transwell migration and invasion tests proved the increase of motility of the TGF-β group and TGEV group cells, confirming that EMT occurred after IPEC-J2 cells were infected with TGEV.

### 3.2. EMT Promoted the Process of E. faecalis Adhering and Invading Cells

The mechanisms of enterococcal infection are adhesion and invasion, just like most bacterial infections. Bacterial adhesion is a key property for bacterial survival and reproduction, as it prevents pathogens from the mechanical removal and grants them a selective advantage over endogenous flora bacteria. Adherence to skin and mucous membranes is usually an early step in the colonization of the host and establishment of infection. Integral host membrane adhesion receptors such as integrins, cadherin, selectin, and CEACAMs are adhesion receptors for many pathogens [7,8]. However, adhering pathogens still face the physical pressure and other host defense mechanisms in extracellular environment (such as cell shedding, complement deposition and antibody labeling). Some bacteria then have evolved molecular strategies to invade target cells for replication and/or spread to other host tissues. Invasion can occur either by direct contact with surface host cell receptors or by direct transposition of bacterial proteins into the cytoplasm of the host cell, which promotes a rearrangement of plasma membrane structures leading to phagocytosis of the pathogen [28]. In this study, *E. faecalis* with MOI of 10, 50 and 100 was incubated with TGEV-infected IPEC-J2 cells, and the dynamic adhesion and invasion analysis showed that TGEV significantly promoted the adhesion and invasion of E. faecalis, where the number of adhering E. faecalis increased by about two to three times and of invaded *E. faecalis* increased by about one to two times, compared with non-EMT cells. In addition, the number of adhering *E. faecalis* was about 10 times that of invading ones. The optimal time point for adhesion and invasion was the concentration of MOI = 100 being incubated for 6 h. Anyway, the adhesion of *E. faecalis* to IPEC-J2 cells in vitro was visually demonstrated by field emission scanning electron microscopy (SEM), which trend was consistent with the experimental results of dynamic adhesion analysis. Similar results were still observed in TGF-β group, which indicated that the adhesion and invasion of *E. faecalis* were significantly improved at the same time of EMT occurred.

The mechanisms of *E. faecalis* adhering and invading cells were tentatively explored, and the results revealed EMT might be the bridge of the TGEV infection and secondary *E. faecalis* involvement. Host cell adhesion molecules, including integrin, cadherin, and members of the immunoglobulin-associated cell adhesion molecule family, are the targets of a bunch of bacteria species during their adhesion and invasion process [29]. Fibronectin-binding protein (EfbA) in *E. faecalis* can bind to FN on the cells [30], and FN can be specifically recognized by integrin-α5 on cells, which is associated with the intracellular actin cytoskeleton and promotes *E. faecalis* to invade host cells. In this study, TGEV infection was confirmed to promote the expression of FN and integrin-α5 in IPEC-J2 cells. This process is similar with *Staphylococcus aureus*, which also expresses fibronectin-binding protein (FNBP-A) binding to ECM-related FN, induces the aggregation of fibronectin-binding integrin receptors, and triggers intracellular signals to induce internalization [31]. Another strategy of bacterial pathogens to cross the host barrier is increasing epithelial or endothelial permeability [32]. Many pathogens target to destroy cell junctions to increase barrier permeability, thereby facilitating the spread of bacteria in the host. In this study, the bacterial migration experiment proved that *E. faecalis* could enter the lower chamber through the intercellular space, and TGEV infection did cause an increase in the number of *E. faecalis* migration, proving that TGEV infection caused the increase of intercellular space and increased cell permeability. Intercellular adhesion junctions are composed of classical cadherin, and classical type I cadherin includes E-cadherin and N-cadherin. E-cadherin contributes to intercellular adhesion and cell integrity and binds to the actin cytoskeleton through the interaction of its cytoplasmic domains between β-catenin and α-catenin. N-cadherin exists at the cell—cell junction and is unique to mesenchymal cells, which are important intercellular junctions that maintain cell integrity and functional stability [33]. In this study, cells in all the three experimental groups have showed similar characteristics with cells in the positive control group in the decreasing expression of E-cadherin and increase expression of N-cadherin, which, together with the increase of the expression of EMT markers, prove that TGEV infection increases cell motility through EMT, and thus promotes the adhesion and invasion of *E. faecalis.*

### 3.3. E. faecalis Alone Could Cause EMT

**Figure A3:**
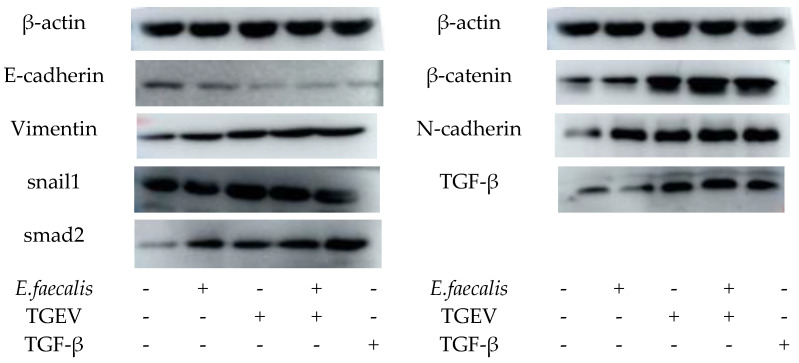
Western blot analysis of the effect of *Enterococcus faecalis* on EMT markers.

**Figure A4:**
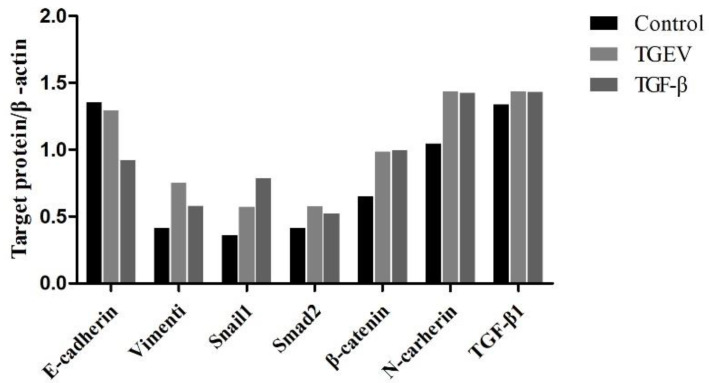
Gray analysis of western blot in Figure 4.

**Figure A5:**
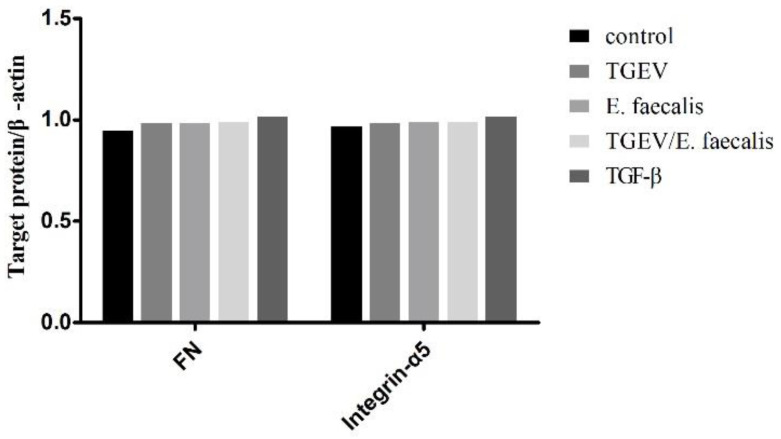
Gray analysis of western blot in Figure 11.

**Figure A6:**
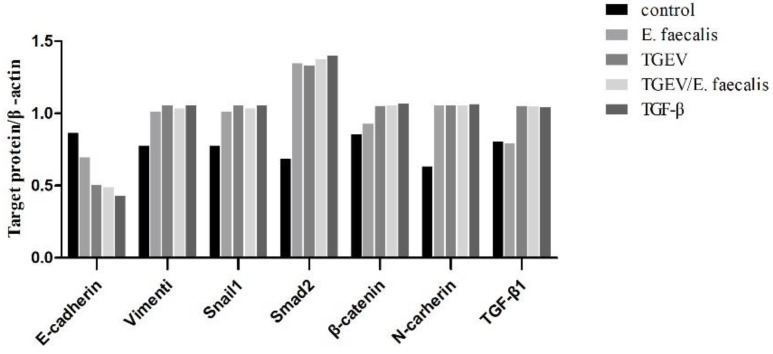
Gray analysis of western blot in Figure A3.

The results also show *E. faecalis* infection alone could cause EMT through the observation of the apparent phenomena, RT-PCR and WB. However, the WB results show that the expression of TGF-β is not up-regulated by *E. faecalis* infection (Figure A3 and Figure A6), which is inconsistent with TGEV infection results, indicating that the pathway of *E. faecalis* to induce EMT is not the TGF-β signaling pathway, and may be different from that caused by TGEV direct involvement of bacterial pathogens in epithelial-to-mesenchymal transformation has been described in a few species, mainly *E. coli* [34], while *H. pylori* [35] and *Klebsiella pneumoniae* [36] have been shown to induce EMT independently. The need for activation of specific pathways that induce the EMT process during certain bacterial infections is maintained by intracellular ROS production and HIF-1a upregulation. *Klebsiella pneumoniae* stimulates A549 cells to produce EMT through hypoxia and oxidative stress [34], while signaling pathway changes in *E. faecalis* during intestinal infection may lead to intracellular stress and tissue/organ damage and may also promote the acquisition of malignant phenotypes.

### 3.4. Attentions to the Potential Hazards of E. faecalis on Swine Were Entailed

Present research of *E. faecalis* infections is mainly concentrated in the field of human medicine, and reports claiming that *E. faecalis* infections in piglets are not noticed, it still stands a good chance of secondary infection by *E. faecalis* following TGEV infection, which significantly increase *E. faecalis* adhesion and invasion of IPEC-J2 cells of piglets. Since the mid to late 1970s, *Enterococcus* has emerged as a major cause of opportunistic pathogens and healthcare-associated infections, most notably urinary tract infections (UTIs) and bacteremia. Being considered to be the most predominant species, *E. faecalis* accounts for over 90% of isolates of such infections. The *Enterococcus* pathogenicity exists in its virulence factors, antibiotic-resistance, which is most verified in the vancomycin-resistant *Enterococcus* (VRE) strains [37,38]. *Enterococcus* might become a major cause of infection or disease, especially in immunocompromised patients, subsequently presenting a challenge to public health [39,40]. *Enterococcus* can transfer antibiotic resistance genes (Args) and genes encoding β -hemolysis, gelatinase, and aggregates, all of which are common virulence characteristics of *Enterococcus*. Moreover, more and more reports on *Enterococcus*’ harms to livestock have emerged, such as bovine mastitis and urethral infection in cats [41,42]. Therefore, there is a reason to believe that the *E. faecalis* infection also has a bad impact on the pig industry, and awareness of its possible epidemic as a potential zoonotic diseases pathogen.

## 4. Materials and Methods

### 4.1. Cells Preparation

The IPEC-J2 cells, TGEV HN-2012 and *E. faecalis* N41 strains were both stored by our laboratory. The IPEC-J2 cells were cultured in Dulbecco’s Modified Eagle’s Medium (DMEM; Gibco, Grand Island, NY, USA) containing 10% FBS (FBS; Gibco, Grand Island, NY, USA) at 37 °C in a humidified atmosphere with 5% CO_2_.

### 4.2. Experiment Design

When the cells grew to 50~70%, TGEV was used to infect the IPEC-J2 cells with MOI of 0.1, 1, and 10, respectively. Cells were cultured with 2% FBS DMEM/F12 nutrient solution. TGF-β was used as a positive inducer in this study because TGF-β is an inducer of EMT in normal mammary epithelial cells [43]. After 24 h starvation of cells, TGF-β was added into DMEM/F12 containing 1% FBS to the final concentration of 10 ng/mL and cultured for 72 h for the experiment.

To evaluate the secondary infection of *E. faecalis*, 5th generation TGEV-infected IPEC-J2 cells were seeded in a pore plate and cultured to continuous monolayer. *E. faecalis* at the middle logarithm was adjusted by Mc Turbidimeter with the concentration of 5 × 10^6^ (MOI = 10), 1 × 10^7^ (MOI = 50) and 5 × 10^7^ CFU/mL (MOI = 100), and then inoculated in IPEC-J2 cells for 2, 4 and 6 h, respectively. For the adhesion assay, both the culture medium and the unattached bacteria were collected and washed by PBS. For the invasion assay, the bacteria were incubated with cells for a certain time after repeated adhesion steps, and then the unadhered cells were collected and washed with PBS three times.

### 4.3. Fluorescent qPCR

The Trizol method was used to extract the RNA of the virus. The extracted RNA was reversely transcribed into cDNA, and the system was reversely amplified. Then, the reverse transcription product was detected by real-time fluorescence quantitative PCR. The reaction system and the amplification program are in Table A1.

To Evaluate the transcriptional and protein levels of EMT markers, successive sub generations to the 5th generation of the TGEV-infected IPEC-J2 cells’ whole RNA was extracted for reverse transcription amplification. Fluorescent primers were designed according to the sequences in GenBank (Table A1). The procedure was carried out by the operation procedure and the recommended reaction system of the qPCR instrument of Bio-rad Company of the United States.

### 4.4. Western Blot

Cells in the well were collected with a cell scraper on ice, and the protein content of each well was quantified. The sample was treated with SDS protein loading buffer, and the protein was fully denatured by boiling for 10min. After 10% SDS-PAGE and a constant flow of 100 mA, the membranes were transferred to 0.22 μm or 0.45 μm (E-cadherin, N-cadherin) PVDF membranes by wetting for 45 min. The membranes were sealed with 5% skim milk powder at room temperature for 2 h. Then they were incubated with corresponding primary antibodies (all from Abways Technology, Inc., Beijing, China) at 4 °C overnight and washed with TBST three times. HRP labeled sheep anti-rabbit secondary antibody (from Proteintech Group, Inc., Wuhan, China) diluted at 1:4000 was incubated at room temperature for 2 h and washed by TBST 3 times, 15 min each. The protein bands were colored with an ECL reagent.

### 4.5. Ghoul Ring Peptide Staining

The 5th generation TGEV-infected IPEC-J2 cells were laid in a 24-well plate with 500 μL per well. An appropriate amount of methanol was used to dissolve the lyophilized polycyclic peptide powder (from US Everbright Inc., Suzhou, China) in the brown tube to prepare 200 T/mL storage liquid. Between each following steps, the cells were washed with PBS three times. The cells were fixed with paraformaldehyde solution on ice for 15 min, and then permeated with PBS solution containing 0.5% Triton-X-100 for 10 min at room temperature. Then, 5 μL fluorescent-labeled phalloidine storage solution was diluted with 200 μL PBS and added to each well, staining for 20 min at room temperature. A quantity of 600 μL DAPI solution were added to each well and incubated at room temperature for 10min. Each well was sealed with a 1mL fluorescence quenching agent (60% glycerin PBS solution) and observed under the fluorescence microscope.

### 4.6. Scratch Test, Transwell Migration, and Transwell Invasion

For the scratch test, cells were inoculated into 6-well plates and grew into consecutive monolayer. Three identical lines were drawn in each well with 200 μL tip. The cell fragments were washed away with PBS, and serum-free nutrient solution was added for further culture. The wound healing was measured, and the wound healing rate was calculated according to the formula:

healing rate = (experimental group 0 h–6 h)/(control group 0 h–6 h).(1)

Transwell cells with the membrane pore size of 8 μm were used in both the Transwell migration assay and invasion assay. In the Transwell cell migration experiment, 200 μL serum-free nutrient solution was first added to the pre-balanced membrane of Transwell cell. IPEC-J2 cells were inoculated with serum-free DMEM/F12 at a concentration of 1 × 10^5^ cells /mL, while 200 μL added to the pre-balanced polyester carbon film in the upper chamber, 500 μL medium containing 10%FBS to the lower chamber. The cells in the upper and lower compartments were removed with cotton swabs, and after fixation with 100% methanol for 10 min, the cells were stained with 0.2% crystal violet, and 5 fields were randomly selected for counting by inverted microscope.

In the Transwell cell invasion experiment, 30 to 50 μL melted matrix gel was added to the upper chamber of Transwell with precooled pipette tip and cured into thin layers at 37 °C for 30 min. The cells were inoculated in the upper chamber, and the medium containing 10% FBS was placed in the lower chamber and incubated at 37 °C for 12 h. After removal of the top cells and fixation with 100% methanol for 10 min, cells at the bottom of the upper chamber were stained with 0.2% crystal violet.

### 4.7. Evaluation of the Adhesion and Invasion of E. faecalis

#### 4.7.1. Tenfold Dilution Method

The TGEV-infected IPEC-J2 cells cultured with *E. faecalis* (see Section 4.2) in 24-well plate were lysed with 500 μL 1% Triton-X-100 and underwent continuous tenfold dilution, while the diluents were coated on BHI AGAR plate. The effective result was the formation of 30–300 colonies on the BHI AGAR plate, which were expressed as the total amount of CFU/mL recovered from each well. Each assay was repeated three times in triplicate.

#### 4.7.2. Indirect Immunofluorescence

The *E. faecalis* N41 strain cultured to the middle logarithm was inoculated with the bacterial concentration adjusted to 5 × 10^7^ CFU/mL (MOI = 100) by the Mc bacterial turbidimeter into the cell slivers filled with IPEC-J2 cells in a 6-well plate for 6 h and washed with PBS three times to remove the non-adherent bacteria. The quantities of 1.5mL tissue fixative for 30 min, 1.5 mL 1% Triton-X-100 for 15 min, and 1.5 mL of 5% bovine serum albumin (BSA) solution for 2 h were added one after another with PBS washing for three times between each step, and slow shaking at 60 r/min was performed to seal. After sealing, 1.5 mL rabbit anti-*E. faecalis* serum (from Abways Technology, Inc., Beijing, China) diluted with 1% BSA solution at 1:500 was added to each well, slowly shaken at 60 r/min for 2 h. Then, this step was repeated with 1.5 mL AF488 labeled sheep anti-rabbit secondary antibody (from Abways Technology, Inc., Beijing, China) diluted at 1:2000 was added. After stained by DAPI and sealed by fluorescence quenching agent (60% glycerin PBS solution), *E. faecalis* adhered to the cells was observed under fluorescence microscope.

#### 4.7.3. Scanning Electron Microscopy

After washing of the non-adherent bacteria step in Section 4.7.2, 1.5 mL electron microscope tissue fixator was added to fix for 1 h. The sample was dehydrated with gradient-concentration acetone solution (10, 30, 50, 70, 90, 100%) on ice for 15 min, and then 100% acetone was replaced. After the sample returned to room temperature, critical point drying was performed with liquid CO_2_. The dried samples were coated with palladium film by sputtering, and then the cell surface status of *E. faecalis* was observed with an accelerated field emission scanning electron microscope (Zeiss Merlin) using an Everhart Thornley SE-detector and an InLens SE-detector at 3 kV.

### 4.8. Data Statistics and Analysis

All the tests were carried out in triplicate, and the results were analyzed by T-test with GraphPad Prism 6 software. (* *p* < 0.05, ** *p* < 0.01, *** *p* < 0.001, **** *p* < 0.0001).

## 5. Conclusions

Overall, continuous TGEV infection of IPEC-J2 cells resulted in morphological changes, decreasing expression of epithelial markers, increasing expression of mesenchymal markers, and enhanced cell motility invasiveness. The phenomena suggest that continuous TGEV infection induces EMT. The TGEV infection changes cell status, which makes a significant increase in the adhesion and invasion potentials of *E. faecalis* to host cells. The increase in the adhesion invasion level of *E. faecalis* is related to the increase in receptor expression level and the change of cell space. *E. faecalis* not only promotes TGEV-induced EMT but also induces EMT itself. In addition, the putative indirect interaction between *E. faecalis* and TGEV is proved by the effect of *E. faecalis* on cytokines. The diversity of bacterial invasion strategies provides new insights into the mechanism of *E. faecalis* pathogenicity.

## Data Availability

The data that support the findings of this study are available from the corresponding author, Zhenzhen G., upon reasonable request.

## Appendix B

**Table A1:** Reaction systems and primers of RT-PCR.

Gene	Primer Sequence (5′-3′)
TGEV N	F-CGCTTGGTAGTCGTGGTGCT
	R-TTGGATTGTTGCCTGCCTCT
β-actin	F-AGATCAAGATCATCGCGCCT
	R-ATGCAACTAACAGTCCGCCT
N-cadherin	F-AGGCAGAAGAGAGACTGGGT
	R-GCTGTACCGCAGAGAAAGGT
E-cadherin	F-CGTATCGGATTTGGAGGGAC
	R-CGAGGAACAAGAGCAGGGTG
β-catenin	F-GCTCTTGTGCGTACTGTCCT
	R-GTCCGTAGTGAAGGCGAACA
Twist	F-CCGGAGACCTAGATGTCATTGT
	R-CCGTCTGGGAATCACTGTC
Snail1	F-TTTTCTCGTCAGGAAGCCCG
	R-AGAGAGTCCCAGATGAGCGT
Smad2	F-GCTGGACGACTACAGCCATT
	R-AGAGGTTTGGAGAACCTGCG
Vimentin	F-GAAGGAGGAAATGGCTCGTC
	R-CTCAGGTTCAGGGAGGAAAAG
Reaction system (20 μL): SYBR Green 10 μL; ddH_2_O 8 μL; upstream primer 0.5 μL; downstream primer 0.5 μL; template 1 μL	
Amplification program (30 cycles from denaturation to extension): pre-denaturation 95 °C 5 min; denaturation 95 °C 30 s; Annealing 59 °C 30 s; extension 72 °C 1 min; determination 72 °C 10 min.	
